# A dataset analysis of digital marketingʼs influence on purchase intentions of millennials and generation Z in Saudi Arabia

**DOI:** 10.1016/j.dib.2024.111045

**Published:** 2024-10-18

**Authors:** Hana Alsaadi, Arwa Wali, Bahjat Fakieh

**Affiliations:** Information Systems Department, King Abdulaziz University, Jeddah, Saudi Arabia

**Keywords:** Digital marketing, Millennials, Generation Z, Purchasing intention

## Abstract

Marketers use digital marketing to influence purchasing intentions and decisions, and have recently focused on millennials and generation Z. Several studies have examined the influence of digital marketing on purchasing intentions, which largely depends on generation and culture, but research in the Arab world is limited, especially in Saudi Arabia. Also, there is a lack of research that predicts purchasing intentions based on digital marketing factors. This research aims to create a dataset that helps to investigate digital marketing factors that influence purchasing intentions among millennials and generation Z in Saudi Arabia. The data are collected by using an online questionnaire that distributed via social media platforms, WhatsApp, X (Twitter), and Telegram. 520 complete responses were obtained during this study. These data can be reused to compare the digital marketing impact among the two generations. The dataset can be a useful resource for local and international companies that target the market segment of millennials and generation Z in Saudi Arabia to develop more effective and attractive marketing strategies.

Specifications Table

**Table:** 

Subject	Data Mining and Statistical Analysis.
Specific subject area	The positive and negative influences of digital marketing factors on the purchasing intentions among individuals from millennials and generation Z.
Type of data	There are three files. The first file is a PDF file that contains the questions of the online survey that was used for data collection. The second file is a CSV file that includes the dataset with numbers and labels. The third file is a PDF file that contains the keys and descriptions of the symbols in the dataset.
Data collection	An online questionnaire was used for data collection. It was conducted from 20/6/2023 to 28/9/2023 through social media platforms. It was designed in two languages, Arabic and English. The target population was millennials and generation Z in Saudi Arabia. The age range for generation Z was from 11 to 26 years, and for millennials, it was from 27 to 42 years. The used sampling technique was convenience sampling. It is a non-probability sampling technique that collects data from the most accessible participants from the target population.
Data source location	Data were collected from millennials and generation Z in Saudi Arabia.
Data accessibility	Repository name: Github Data identification number: https://doi.org/10.5281/zenodo.13372132 Direct URL to data: https://github.com/Hana0111/Digital-Marketing.git Instructions**:** Data are available in the provided link.
Related research article	None.

## 1 Value of the Data

• These data include demographic variables and the digital marketing factors that can positively or negatively influence the purchasing intentions of individuals from millennials and generation Z. This could be useful to understand the impact of different digital marketing factors on the purchasing intentions of individuals of those generations.
• These data can help governments, decision makers and local and international marketers to get better insight into the influential factors on the purchasing intentions of millennials and generation Z in Saudi Arabia which could lead to better decisions.
• The dataset would provide useful resources for conducting cross-cultural studies across different countries. It can be compared with studies applied to similar markets in the same region, such as the UAE or Qatar, and with studies conducted on different cultural markets, such as Jordan, Egypt, Turkey, or European countries.
• These data can be used to predict and classify purchasing intentions (positive, negative, or neutral) based on digital marketing factors.

## 2 Background

The main objective for collecting the dataset [1] was to get a better understanding of digital marketing factors that influence purchasing intentions of millennials and generation Z in Saudi Arabia. It focuses on those factors that can positively influence purchasing intentions, dominate as DM drivers, and those factors that have negative influence, dominate as DM obstacles. The second objective was to predict purchasing intentions based on digital marketing factors. Researchers can use it to understand the impact of digital marketing on purchasing intentions. Also, they can use it to compare this impact among individuals of the two generations. Moreover, they can use it to study whether this impact differs among young and older individuals from the same generation. Furthermore, it can be used to classification purchasing intentions according to digital marketing factors.

## 3 Data Description

There are three files related to the data. First, there is a PDF file that includes the questions of the online survey that was used as the data collection tool, this file is called “Questions of online questionnaire”. The original data are included in .CSV file called “Digital marketing dataset”. Also, there is a PDF file that contains the keys and descriptions of the dataset's symbols, this file is called “Dataset Keys”. The CSV file includes the demographic variables of the individuals. Also, it contains a set of digital marketing factors with its impact of individuals purchasing intentions (positive, negative, or neutral). Furthermore, it involves class labels that describe the impact of digital marketing on individuals’ purchasing intentions, either positive, negative, or neutral intentions. This file includes around 520 records.

The data are collected by using an online questionnaire. The Internet penetration rate in Saudi Arabia has been increasing to reach 98.1 % in 2021 (the period of collecting the data), and it is continuously increasing [2]. In Saudi Arabia, there are three main telecommunication companies that provide a free access to the social media platforms in most of their plans. Due to that, different social media platforms were used to distribute the questionnaire to the targeted population. These platforms were WhatsApp, X (previously Twitter), and Telegram. The ease of access to these platforms supported the dissemination of the online questionnaire among the targeted population.

**Fig. 1:**
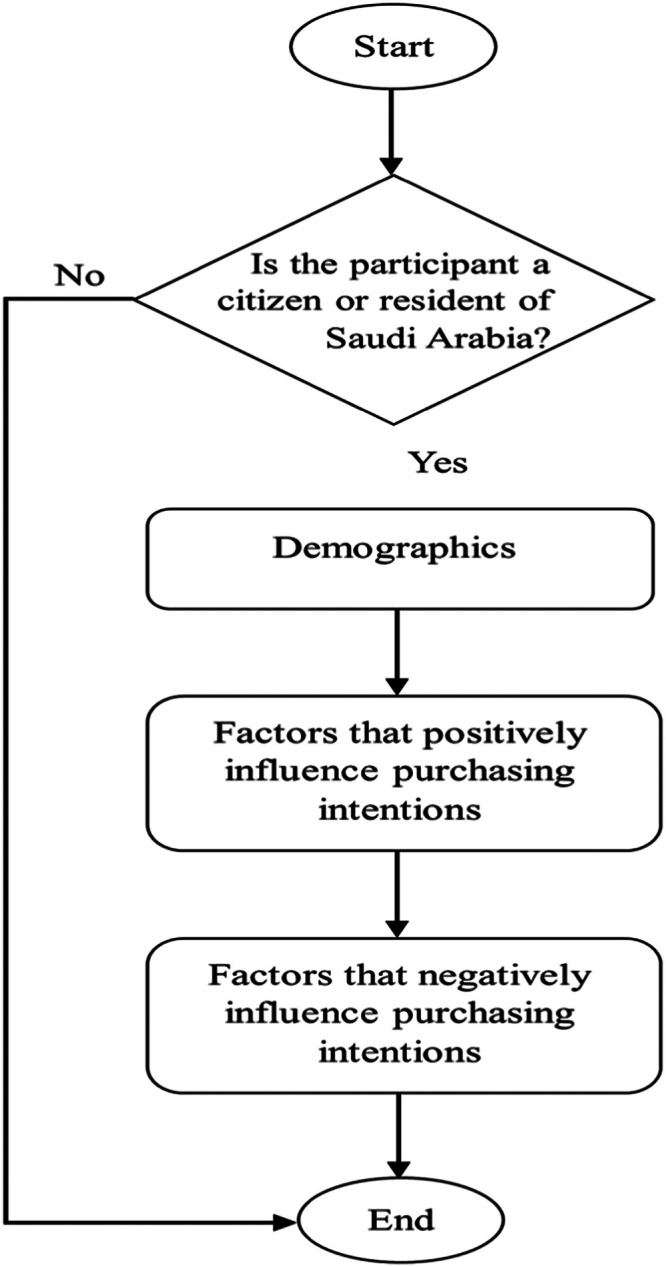
Questionnaire flow.

The questionnaire was designed in two languages, Arabic and English, since it targets individuals in Saudi Arabia. It consisted of 34 closed-ended and Likert scale questions, divided into three main sections. All the Likert scale questions have range values of 1 to 3, which indicate whether the factor has a positive, negative, or neutral influence on the purchasing intention. Fig. 1 shows the flow of the questionnaire.

The first section was about demographic variables and online shopping habits. It consisted of 10 closed-ended questions related to age, gender, education, marital status, number of children, location, income, average hours of mobile phone use per day, monthly shopping rates, and behaviors when seeing digital ads.

The second section was about factors that positively influence purchasing intentions: digital ad format, marketing channel, ad content and language, scarcity phrases, promotional offers phrases, and advertising endorsers. It also included repeated ad exposure, eWOM, and influencer marketing. This section consisted of 17 closed-ended and Likert scale questions.

The final section was related to factors that may negatively influence purchasing intentions. These factors include shipping costs, delivery time, strict return policies, cash on delivery, and lack of online reviews. This section consisted of 5 Likert scale questions.

The target population was millennials and generation Z in Saudi Arabia. The age range of generation Z was considered to be from 11 to 26 years, and of millennials from 27 to 42 years [3]. Each generation is classified into two subcategories which are younger individuals and older individuals. The purpose of this separation is to facilitate data analysis and support achieving the objectives of data collection. The age range of younger generation Z was from 11 to 18 years old, and the older generation Z was aged from 19 to 26 years old. The younger millennials were aged from 27 to 34 years old, and the older millennials aged from 35 to 42 years old.

Convenience sampling was used as sampling technique. Thus is a non-probability sampling technique that collects data from the most available and accessible participants from the target population. It can be applied in qualitative and quantitative research and is helpful in research into very large populations when it is impossible to apply randomization [4].

Finally, there is a question that identifies the impact of digital ads on purchasing intentions.

First, a pilot questionnaire was applied, which is a technique for pre-testing the data collection tool [5]. After this, the questionnaire was distributed to the target population via social media.

Table 1 presents the sample distribution of the target population based on demographic variables, where the sample size is *N* = 520. The individuals of each generation are divided into two groups, younger and older individuals. The purpose of this separation is to simplify the analysis and investigate whether DM factors have different influences on younger and older individuals from the same generation. Most of the respondents were from generation Z (59.23 %). The sample included more older individuals (36.15 %) than younger individuals (23.08 %) from this generation. Among the millennials, the sample of younger individuals (22.12 %) was larger than the sample of older individuals (18.65 %). The sample included more females (46.35 % from generation Z and 29.62 % from millennials) than males (12.88 % from generation Z and 11.15 % from millennials). In the sample from generation Z, most were single (56.92 %), whereas among the millennials, most were married (27.31 %). Regarding the location, most of the respondents were from The Western Region (57.69 %), while those from The Northern Area were the fewest (4.04 %).

**Table 1:** Sample distribution based on demographic variables (*N* = 520).

Demographic variables	Younger generation Z n (%)	Older generation Z n (%)	Younger millennials n (%)	Older millennials n (%)
**Gender**				
*Female*	108 (20.77 %)	133 (25.58 %)	88 (16.92 %)	66 (12.69 %)
*Male*	12 (2.31 %)	55 (10.58 %)	27 (5.19 %)	31 (5.96 %)
**Marital Status**				
*Single*	119 (22.88 %)	177 (34.04 %)	52 (10 %)	18 (3.46 %)
*Married*	1 (0.19 %)	11 (2.12 %)	63 (12.12 %)	79 (15.19 %)
**Location**				
*The Central Region*	20 (3.85 %)	33 (6.35 %)	31 (5.96 %)	17 (3.27 %)
*The Eastern Province*	16 (3.08 %)	16 (3.08 %)	10 (1.92 %)	9 (1.73 %)
*The Northern Area*	3 (0.58 %)	11 (2.12 %)	6 (1.15 %)	1 (0.19 %)
*The Southern Area*	10 (1.92 %)	20 (3.85 %)	11 (2.12 %)	6 (1.15 %)
*The Western Region*	71 (13.65 %)	108 (20.77 %)	57 (10.96 %)	64 (12.31 %)
*Total*	120 (23.08 %)	188 (36.15 %)	115 (22.12 %)	97 (18.65 %)

## 4 Experimental Design, Materials and Methods

### 4.1 Data preprocessing

Data preprocessing aims to enhance raw data quality [6]. The techniques used were data cleaning, and data transformation.

Data Cleaning involves handling missing and inconsistent data [6]. Removing Arabic characters and handling missing values were the subtasks in this stage. The questionnaire was distributed in two languages, but Arabic text was removed to facilitate data processing using Visual Basic for Applications (VBA), a version of Visual Basic for Microsoft Office applications [7]. Then, the missing values were handled.

Data Transformation adjusts data presentation to a suitable input format for data mining models [8]. In this stage, numeralization and attribute construction were applied. Numeralization means transforming categorical data into numerical data [6,8]. Sequential encoding is a technique that assigns a unique numerical index to each categorical value [8]. It was applied to the attributes, and then attribute construction was applied. This means constructing new attributes from the existing attributes [9]. It was applied to attributes that contained many values, such as ‘attractive digital ad format’, ‘digital ad channels’, and ‘behaviors when seeing the ads’. For example, the ‘attractive ad type’ attribute had a combination of five values, so five new attributes were constructed.

### 4.2 Data analysis

**Fig. 2:**
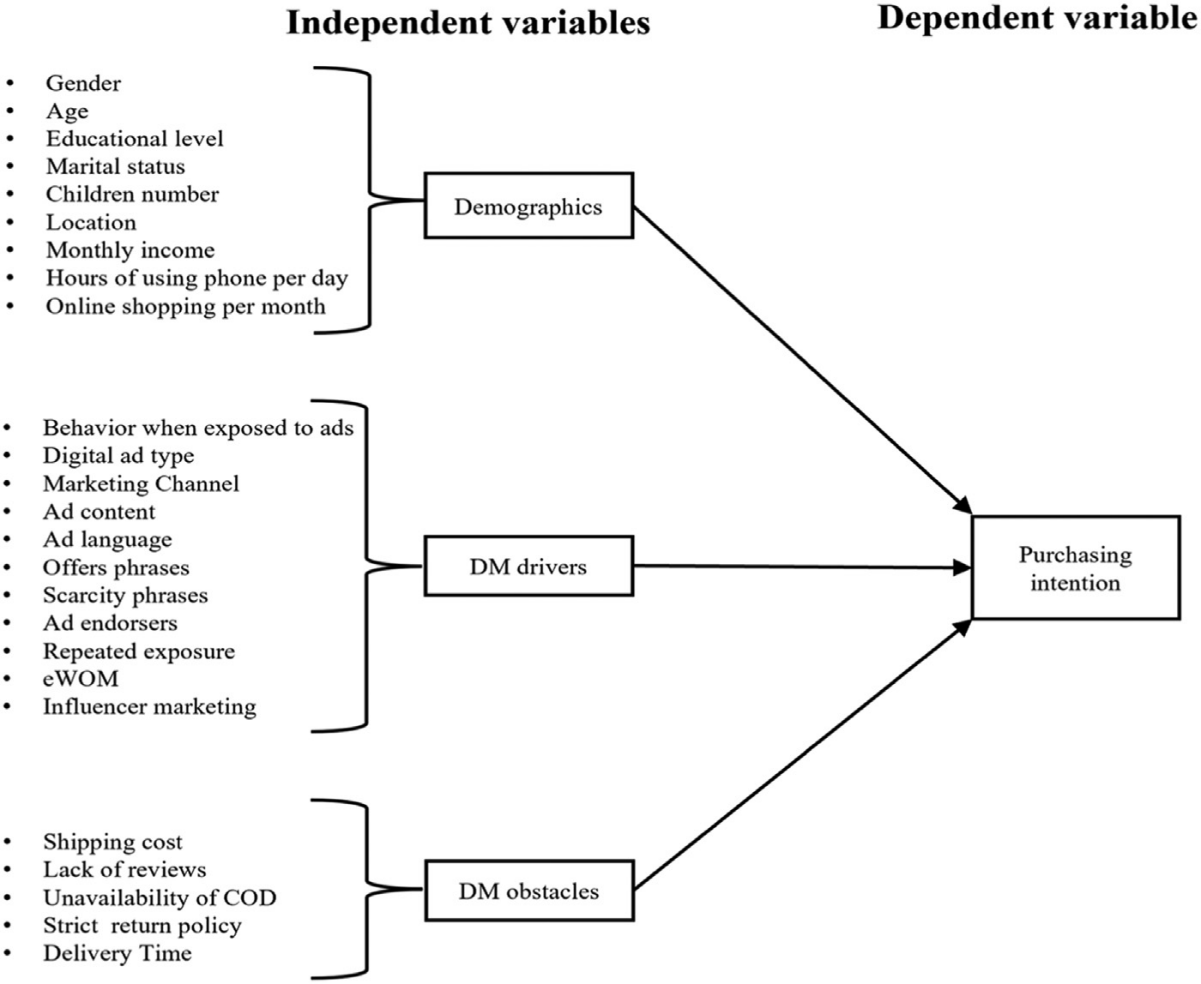
Overview of independent and dependent variables.

Microsoft Excel was used for data analysis. Fig. 2 provides an overview of thefactors understudy in this research.

In the following, some descriptive statistics about the characteristics of the respondents are provided. It includes the average hours of using mobile phones for younger and older individuals of both generations. Also, the rate of online shopping per month for each of the four categories is presented. Moreover, the attractive formats of presented digital advertising for both generations are described. The attractive and preferable marketing channels to present digital advertising that targets these categories from the perspectives of the individuals of both generations.

**Fig. 3:**
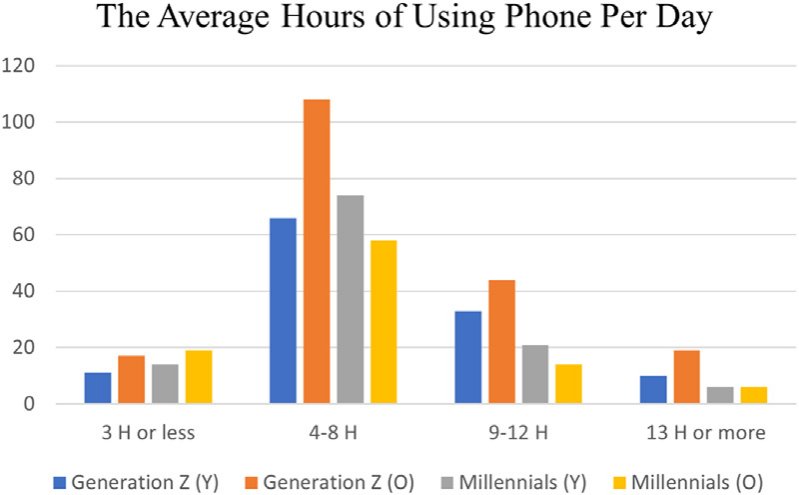
The average hours of using phone per day.

The average hours of using the phone per day by younger and older individuals of both millennials and generation Z is explained in Fig. 3. It is obvious that the daily average hours of using phones for most individuals of all generations is four to eight hours. Regarding the younger individuals from both generations, few individuals use their phones for three hours or less a day, while even fewer use them for an average of 13 h or more a day. A small number of older individuals of generation Z reach 13 h or more as an average daily hours of usage, and the minority of this category record an average of three hours or less per day. The lowest average hours of using the phone among older individuals of millennials are 9–12 h and 13 h or less, respectively.

**Fig. 4:**
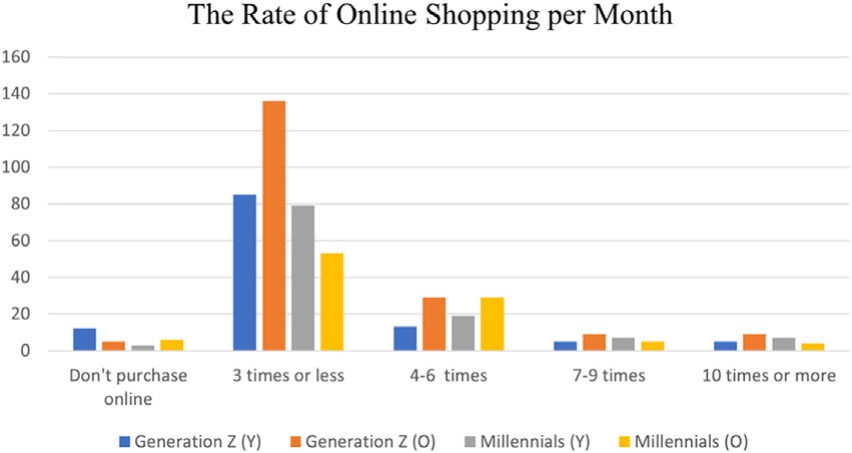
The rate of online shopping per month.

The monthly rate of online shopping among the younger and older individuals of millennials and generation Z is presented in Fig. 4. It appears that the majority of all generation categories tend to do online shopping up to three times per month. Few individuals of younger generation Z and older millennials do online shopping 7–9 times monthly, and few others do it ten times or more per month. In regard to older generation Z and younger millennials, the minority do not do online shopping.

**Fig. 5:**
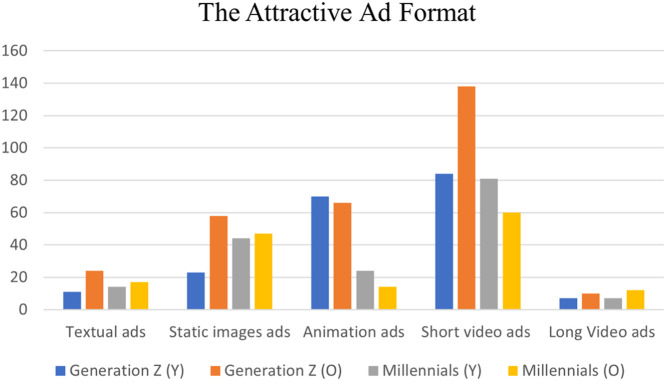
The attractive Ad format.

The dataset considers five of the popular digital advertising formats, which are textual ads, static image ads, animation ads, short video ads (not exceeding 15 s), and long video ads (more than 15 s). Fig. 5 shows the favorite ad formats for each of the four categories. Short video ads are the most favorite form for all categories. However, long video ads are the advertising form that has the lowest level of attractiveness among individuals of both generations. The textual ads form has a low attractiveness level as well. Regarding the animation and static ads, the animation ads seem to be more attractive for both younger and older individuals of generation Z, while the static image ads are more attractive for the younger and older individuals of millennials.

**Fig. 6:**
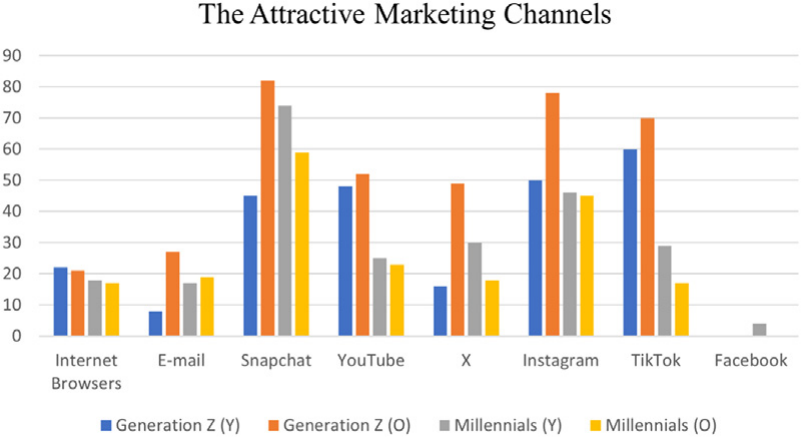
The attractive marketing channels.

The dataset focuses on the common channels that are used to deliver digital advertising. These platforms are Internet browsers, e-mail, Snapchat, YouTube, X, Instagram, TikTok, and Facebook. Fig. 6 illustrates the attractiveness level of using these channels to deliver digital advertising to millennials and generation Z. As shown in the graph, TikTok is the most attractive channel for younger individuals of generation Z. At the same time, Snapchat is the most attractive channel for individuals of the other generation categories. Facebook represents the lowest level of attractiveness for most respondents of all generation categories except few individuals of younger millennials. For younger individuals of generation Z, X, and e-mail have low levels of attractiveness compared to other channels. Regarding the older individuals of generation Z and younger individuals of millennials, e-mail, and internet browsers have low levels of attractiveness. For older individuals from millennials, e-mail, X, Internet browser, and TikTok have approximately the same level of attractiveness.

## Limitations

The main limitation of this research is the sample size. There were some difficulties in data collection, especially from men. Also, there was difficulty in reaching individuals from different cities. There is a bias in the data regarding the gender. Most of the responses were from females. It would be good to collect data from different cities in Saudi Arabia in the future. Moreover, collecting data from a larger sample or different cultures will be better.

Another limitation is the method that was used to distribute the online questionnaire, which was social media platforms. This method caused some biases in the collected data, such as gender, and location. In future studies, it will be better to target specific people directly, which will help reach almost an equal number of respondents in each category and avoid biases in the dataset.

## Ethics Statement

This research was reviewed by the ethics committee at King Abdulaziz University. The committee waived this study from obtaining the approval as the study does not expose any personal information nor include any dangerous or harming activities. However, it was advised that the distributed survey must start by a consent that gives participants the complete right to decide to participate or not in the study, as well as the complete choice to answer or skip any questions, which was included at the beginning of the distributed online questionnaire.

These data were collected from individuals in Saudi Arabia through social media. A written informed consent was obtained from all participants before they contributed in the study. Regarding the participants under 18 years old, they were asked to send consent from their parents or guardians via email to ensure that they agreed for their child to participate in the study.

## CRediT Author Statement

**Hana Alsaadi:** Conceptualization, Methodology, Software, Formal analysis, Investigation, Resources, Data curation, Writing—original draft preparation. **Arwa Wali:** Conceptualization, Methodology, Validation, Writing—review and editing, Supervision, project administration. **Bahjat Fakieh:** Conceptualization, Methodology, Validation, Writing—review and editing, Supervision, project administration*.*

## Data Availability

GithubDigital-Marketing (Original data).
